# PDX1 in early pregnancy is associated with decreased risks of gestational diabetes mellitus and adverse pregnancy outcomes

**DOI:** 10.3389/fendo.2025.1486197

**Published:** 2025-05-08

**Authors:** Qian Zhang, Qing-qing Zhang, Shu-qin Dong, Xia Liu, Jing Wei, Kai Li, Yu Lu

**Affiliations:** ^1^ Department of Endocrinology, The Affiliated Taizhou People’s Hospital of Nanjing Medical University, Taizhou School of Clinical Medicine, Nanjing Medical University, Taizhou, China; ^2^ Department of Obstetrics and Gynecology, The Affiliated Taizhou People’s Hospital of Nanjing Medical University, Taizhou School of Clinical Medicine, Nanjing Medical University, Taizhou, China; ^3^ Key Laboratory of Human Functional Genomics of Jiangsu Province, Nanjing Medical University, Nanjing, China

**Keywords:** pancreatic duodenal homeobox-1, gestational diabetes mellitus, adverse pregnancy outcomes, early pregnancy, mid-pregnancy

## Abstract

**Aim:**

To investigate the association of pancreatic duodenal homeobox-1 (PDX1) in early pregnancy with the risks of gestational diabetes mellitus (GDM) and adverse pregnancy outcomes.

**Methods:**

A total of 231 pregnant women were recruited at their initial antenatal care visit during 8-12 gestational weeks in this study. The 75g OGTT was performed during 24-28 gestational weeks. Blood samples were collected to measure PDX1 levels. Participants were followed throughout their pregnancy to monitor for the development of GDM and adverse pregnancy outcomes. The odds ratio (OR) was used to assess the risks of GDM and adverse pregnancy outcomes.

**Results:**

Pregnant women in the GDM group had higher levels of HOMA-IR and TyG index, and lower PDX1 levels both in early and mid-pregnancy (P<0.05), but had lower HOMA-β levels only in mid-pregnancy (P<0.05). PDX1 in early pregnancy was negatively correlated with FPG, 2h PG, HOMA-IR, and TyG, while positively correlated with HOMA-β in mid-pregnancy (P<0.05). The adjusted analysis showed that elevated PDX1 levels in early pregnancy were associated with reduced risks of GDM (aOR 0.287, 95%CI 0.130-0.636, P=0.002), macrosomia (aOR 0.249, 95%CI 0.076-0.811, P=0.021) and composite adverse pregnancy outcomes (aOR 0.496, 95%CI 0.256-0.960, P=0.037).

**Conclusion:**

Elevated PDX1 in early pregnancy was associated with decreased risks of GDM and adverse pregnancy outcomes.

## 1 Introduction

Gestational diabetes mellitus (GDM), a form of diabetes, is identified during pregnancy in women who did not have diabetes before pregnancy. It is generally diagnosed at 24-28 weeks of pregnancy using an oral glucose tolerance test (OGTT) (1). Insulin resistance (IR) and pancreatic β-cell dysfunction are thought to be important mechanisms in the development of GDM (2). In fact, GDM is a common complication in pregnant women, with recent data indicating a prevalence of 20.8% in Southeast Asian women (3) and 21.1% in Chinese women (4). GDM can significantly and seriously impact both maternal and fetal health. It is linked to increased adverse pregnancy outcomes, including pre-eclampsia, preterm birth, macrosomia, and prenatal depressive symptoms (5). Women diagnosed with GDM face a risk of developing diabetes over 20 times higher than those without GDM (6). Additionally, the risks of cardiovascular diseases, hypertension, kidney disease, hyperlipidemia, and incident dementia all increase from one to six times, and these risks escalate with the duration of delivery (6, 7). Infants of mothers with GDM frequently experience hypoglycemia and jaundice, and they face a higher likelihood of becoming obese and developing type 2 diabetes later in life. Given the potential harms associated with GDM, it is imperative to identify GDM as early as possible.

Pancreatic duodenal homeobox-1 (PDX1) is a nuclear transcription factor expressed in both endocrine and exocrine cells before embryo maturation. However, as the pancreas matures, its expression becomes predominantly restricted to β-cells (8, 9). It plays a pivotal role in the development of the pancreas, the differentiation of β-cells, and the preservation of mature β-cell functions. PDX1 can bind to and activate the promoter of the insulin gene expression, thereby increasing the synthesis of insulin and maintaining glucose homeostasis (10). Existing studies indicate that the decreasing of PDX1 expression leads to abnormalities in blood glucose regulation, thereby impacting the onset and progression of diabetes (11, 12). Based on the role of PDX1 in glucose regulation, PDX1 may be implicated in glucose metabolic disorders during pregnancy.

In this prospective study, we explored the association of PDX1, GDM, and adverse pregnancy outcomes in Chinese women to identify early prediction and prevention strategies for GDM and adverse pregnancy outcomes.

## 2 Materials and methods

### 2.1 Study population and design

**Figure 1:**
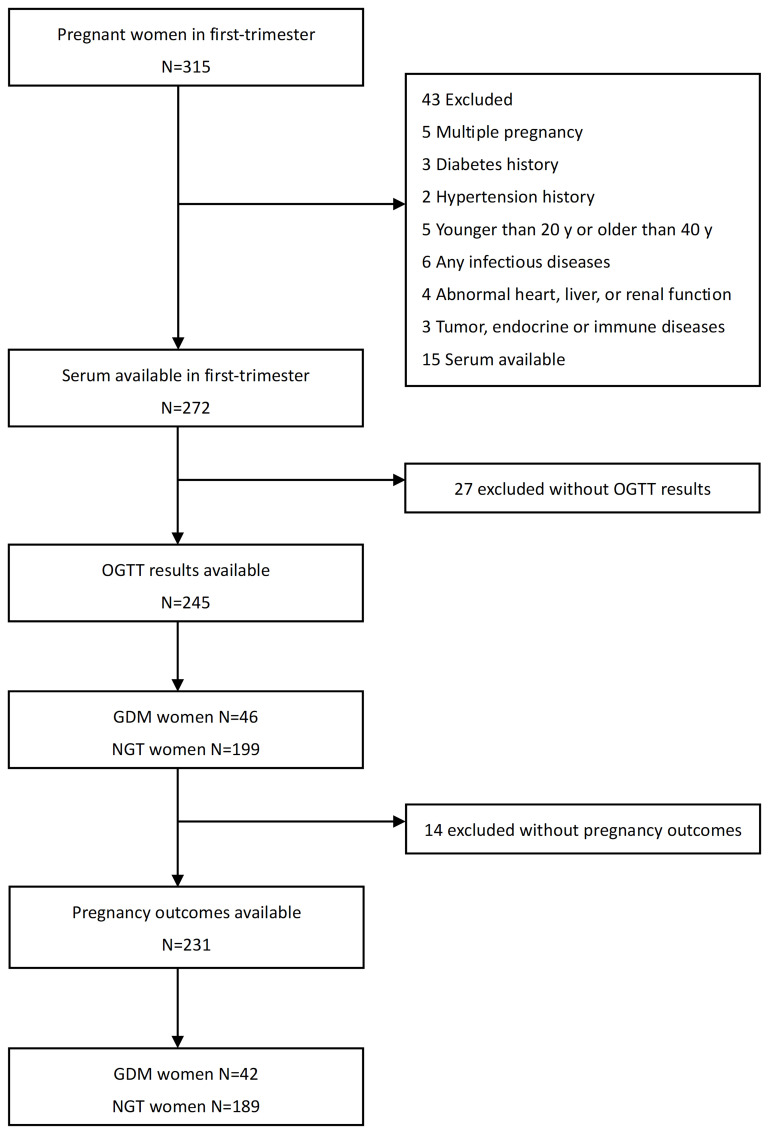
Flow chart of included subjects.

The study cohort and methods were described previously (13). Briefly, from October 2020 to March 2022, we established a preconception cohort of pregnant women based on a screening and management system in Taizhou People’s Hospital. We initially recruited 315 singleton pregnant women aged 20 to 40 years old during their first prenatal examination in the hospital at 8-12 gestational weeks. Individuals with a history of abnormal glucose tolerance, diabetes, hypertension, polycystic ovary syndrome, hyperthyroidism, hypothyroidism, malignancies, autoimmune diseases, or severe cardiac, hepatic, or renal dysfunction were excluded. They were followed up from the initial prenatal examination until the completion of delivery. The OGTT was conducted during 24-28 weeks of pregnancy to diagnose GDM based on its results. Finally, 231 pregnant women were included in this study, 42 were diagnosed with GDM (GDM group), while 189 had normal glucose tolerance (non-GDM group). The flow chart is shown in **Figure 1**. This study complied with the Helsinki Declaration and was approved by the Institutional Ethics Committee of Taizhou People’s Hospital.

### 2.2 Definition of GDM and adverse pregnancy outcomes

According to the 75g OGTT results, GDM was diagnosed based on World Health Organization 2013 criteria. Any one of the following criteria needs to be met: (1) fasting plasma glucose (FPG) ≥ 5.10 mmol/L; (2) 1-hour postprandial blood glucose (1h PG) ≥ 10.00 mmol/L; (3) 2-hour postprandial blood glucose (2h PG) ≥ 8.50 mmol/L (14).

Adverse pregnancy outcomes in this study were defined as pathological pregnancy and abnormal pregnancy, including pre-eclampsia, fetal growth restriction (FGR), preterm birth, macrosomia, and neonatal respiratory distress syndrome (NRDS). The composite adverse pregnancy outcomes included any one or a combination of the adverse events mentioned above. Pre-eclampsia was characterized by a systolic blood pressure (SBP) ≥140mmHg and/or a diastolic blood pressure (DBP) ≥90mmHg after 20 weeks of pregnancy. FGR was considered as ultrasonographic estimated fetal weight (EFW) or abdominal circumference (AC) below the 10th percentile of the normal gestational age (15). Preterm birth was defined as childbirth taking place between the 24th and 37th weeks of pregnancy. When a newborn’s weight exceeded 4000g, it was classified as macrosomia. The diagnosis of NRDS is based on symptoms of respiratory distress, oxygen levels in the blood, and abnormal results from chest X-rays by professional pediatricians (16, 17).

### 2.3 Data collection and measurement of serum PDX1

A standardized procedure was performed since the initial antenatal care visit to the hospital. A questionnaire was administered to collect the information, including height, pre-pregnancy weight, smoking and alcohol habits, parity, family history of diabetes, and history of metabolic disorders. The pre-pregnancy BMI (Body Mass Index) was calculated by dividing pre-pregnancy weight(kg) by height squared(m2).

Blood samples were collected in the morning after at least 8 hours of fasting and analyzed in the hospital’s central laboratory. The results of the OGTT were recorded at weeks 24-28 of pregnancy from the electronic medical record system. The homeostatic model was used to assess insulin resistance (HOMA-IR=FIns×FPG/22.5), and insulin beta cell function (HOMA-β=20×Fins/(FPG-3.5)). Triglyceride and glucose (TyG) index was also calculated to assess insulin resistance (TyG index = Ln [fasting triglyceride (mg/dL)×fasting glucose (mg/dL)/2]) (18, 19). Serum aliquots were preserved for further analysis. For short-term storage, serum samples were maintained at 2–8°C for a maximum of 24 hours before being aliquoted and transferred to −80°C for long-term storage (up to 2 years). Hemolytic samples were excluded from the analysis to ensure data reliability. The enzyme-linked immunosorbent assay (ELISA) kit (Shanghai Zhenke Biology Co., Ltd., China) was used to quantify serum PDX1 levels. To minimize variability, each sample was measured in duplicate within the same analytical session, and the average value was used for further analysis. Intra- and inter-assay coefficients of variation (CVs) were controlled at 10% and 15%, respectively, by calibrating the equipment before each session and using standardized protocols across all measurements.

### 2.4 Statistical analysis

The analysis of statistical data and the creation of figures were realized by SPSS 26.0 (IBM SPSS Inc, Chicago, IL, USA) and GraphPad Prism 9.5. For variables following a normal distribution, the Student’s t-test was used to calculate their mean and standard deviation (SD), while for variables not following a normal distribution, the Mann–Whitney test was used to measure their median and interquartile range. The Chi-square test was used for categorical variables between two groups, and percentages were calculated for categorical variables. We used Spearman correlation analysis to assess the relations of PDX1 with glucose metabolic indicators. Logistic regression analysis was performed to examine the associations of PDX1 with GDM and adverse pregnancy outcomes. Receiver operating characteristic (ROC) curves measured by R language were used to assess the predictive ability of PDX1 for GDM. A p-value less than 0.05 was regarded as statistically significance.

## 3 Results

### 3.1 Characteristics of participants in GDM and non-GDM groups

In this study, 231 patients were recruited with an average age of 28 years and an average pre-pregnancy BMI of 22.11 kg/m². Participants were divided into two groups according to the results of OGTT. 42 pregnancies occurred GDM (GDM group), while the other 189 pregnancies exhibited glucose tolerance within the normal range (non-GDM group). There was a significant difference in age between the two groups, with the GDM group being older (30 vs 28 years, P=0.029). No statistical differences between the two groups in the number of male fetuses.

Patients in the GDM group had elevated FPG levels in the first trimester and second trimester (4.75 vs 4.63 mmol/L, 5.12 vs 4.36 mmol/L, P<0.05, respectively). Regardless of early or mid-pregnancy, HOMA-IR, TyG index and triglycerides (TG) were all higher in the GDM group (P<0.05). However, HOMA⁃β levels only showed lower levels in the GDM group in mid-pregnancy (148.97 vs 198.59, P=0.002). Additionally, PDX1 was lower in the GDM group in both two stages (123.21 vs. 132.15 pg/mL, P=0.013; 81.65 vs. 96.77 pg/mL, P<0.001, respectively, **Table 1**). Furthermore, from early pregnancy to mid-pregnancy, HOMA-IR, TyG index, and TG were all significantly increased in both GDM and non-GDM groups, while PDX1 was decreased (P<0.05) (**Supplementary Table 1**).

**Table 1:** Characteristics of participants in GDM and non-GDM groups.

Index	All	GDM group	non-GDM group	Z/χ2	*P* value
	N=231	N=42	N=189		
Age (year)	28 (26,30)	30 (26,32)	28 (26,30)	-2.187	0.029
Preconception BMI (kg/m²)	22.11 (20.75,24.22)	23.23 (21.14,26.03)	22.04 (20.63,24.02)	-1.858	0.063
Family history of diabetes,n (%)	10 (4.33)	3 (7.14)	7 (3.70)	0.327	0.568
Smoking exposure,n (%)	9 (3.90)	3 (7.14)	6 (3.17)	2.699	0.100
Alcohol consumption,n (%)	18 (7.79)	5 (11.90)	13 (6.88)	0.021	0.885
Male fetuses,n (%)	121 (52.38)	23 (54.76)	98 (51.85)	0.117	0.733
Early pregnancy					
FPG (mmol/L)	4.65 (4.42,4.92)	4.75 (4.47,5.23)	4.63 (4.42,4.88)	-2.153	0.031
HOMA-β	113.87 (84.84,157.93)	110.26 (83.52,188.32)	114.42 (83.82,156.82)	-0.373	0.709
HOMA-IR	1.44 (1.02,1.85)	1.72 (1.07,2.34)	1.39 (1.02,1.79)	-2.341	0.019
TyG index	8.52 (8.36,8.79)	8.76 (8.52,9.08)	8.49 (8.31,8.75)	-4.623	<0.001
TC (mmol/L)	4.38 (4.03,4.94)	4.41 (4.02,5.06)	4.37 (4.04,4.89)	-0.867	0.386
TG (mmol/L)	1.36 (1.14,1.74)	1.62 (1.35,2.30)	1.33 (1.09,1.64)	-4.082	<0.001
HDL-C (mmol/L)	1.61 (1.42,1.82)	1.61 (1.43,1.85)	1.61 (1.42,1.81)	-0.416	0.645
LDL-C (mmol/L)	2.45 (2.18,2.85)	2.47 (2.15,2.86)	2.44 (2.18,2.85)	-0.870	0.385
PDX1 (pg/ml)	131.15 (118.29,142.96)	123.21 (108.38,139.78)	132.15 (119.35,143.10)	-2.48	0.013
SBP (mmHg)	114 (107,120)	117 (110,125)	113 (107,120)	-1.891	0.059
DBP (mmHg)	71 (65,77)	72 (65,79)	70 (65,76)	-0.82	0.412
Mid-pregnancy					
FPG (mmol/L)	4.42 (4.18,4.68)	5.12 (4.66,5.41)	4.36 (4.15,4.59)	-7.241	<0.001
1h PG (mmol/L)	7.54 (6.68,8.65)	9.88 (8.46,10.89)	7.33 (6.40,8.24)	-7.245	<0.001
2h PG (mmol/L)	6.75 (6.05,7.45)	8.53 (7.19,9.77)	6.60 (5.92,7.17)	-6.929	<0.001
HOMA-β	191.53 (132.65,285.65)	148.97 (112.21,209.16)	198.59 (140.82,298.03)	-3.058	0.002
HOMA-IR	1.66 (1.29,2.25)	2.27 (1.65,3.62)	1.57 (1.22,2.12)	-4.628	<0.001
TyG index	9.00 (8.80,9.25)	9.33 (9.03,9.68)	8.95 (8.77,9.19)	-5.417	<0.001
TC (mmol/L)	5.63 (5.14,6.29)	5.90 (5.06,6.37)	5.56 (5.15,6.27)	-1.011	0.312
TG (mmol/L)	2.32 (1.87,2.90)	2.70 (2.23,4.07)	2.21 (1.85,2.75)	-3.698	<0.001
HDL-C (mmol/L)	1.90 (1.72,2.13)	1.91 (1.72,2.10)	1.90 (1.73,2.14)	-0.107	0.915
LDL-C (mmol/L)	3.23 (2.87,3.61)	3.27 (2.85,3.66)	3.23 (2.88,3.58)	-0.236	0.813
PDX1 (pg/ml)	92.64 (74.93,109.58)	81.65 (71.34,92.75)	96.77 (77.43,111.56)	-3.523	<0.001
SBP (mmHg)	113 (108,120)	115 (109,122)	112 (108,119)	-1.371	0.170
DBP (mmHg)	70 (65,76)	71 (66,76)	70 (64,76)	-0.402	0.687

GDM, gestational diabetes mellitus; BMI, body mass index; FPG, fasting plasma glucose; PDX1, pancreatic duodenal homeobox-1; TyG index, triglyceride and glucose index; 1h PG, 1-hour postprandial blood glucose; 2h PG, 2-hour postprandial blood glucose; SBP, systolic blood pressure; DBP, diastolic blood pressure; TC, total cholesterol; TG, triglycerides; HDL-c, high-density lipoprotein cholesterol; LDL-c, low-density lipoprotein cholesterol.

### 3.2 Characteristics, incidence of GDM and adverse pregnancy outcomes in two groups categorized by PDX1 in early pregnancy

We divided the participants into two groups based on the median PDX1 level in early pregnancy (131 pg/mL): a low PDX1 group (n=115) and a high PDX1 group (n=116). The prevalence of GDM was significantly higher in the low PDX1 group compared to the high PDX1 group (26.09% vs 10.34%, P=0.002). The low PDX1 group exhibited higher levels of FPG (4.57 vs 4.29 mmol/L, P<0.001) and HOMA-IR (1.83 vs 1.57, P=0.005), while HOMA-β levels were lower (175.27 vs. 207.16, P=0.022; **Table 2**). Pregnant women in the high PDX1 group had a lower incidence of preterm birth (11.30% vs. 4.31%, P=0.047), macrosomia (11.30% vs. 3.45%, P=0.022), and composite adverse pregnancy outcomes (28.70% vs. 16.38%, P=0.025). When grouped according to fetal sex, it was found that PDX1, FPG, HOMA-IR, HOMA-β, and other metabolic indicators and adverse pregnancy outcomes were not statistically different between the two groups (**Supplementary Table 2**).

**Table 2:** Groups Categorized by PDX1 in early pregnancy.

Index	All	PDX1<131pg/mL	PDX1≥131pg/mL	Z/χ2	*P* value
	N=231	N=115	N=116		
Age (year)	28 (26,30)	28 (26,30)	28 (26,30)	-0.512	0.608
Preconception BMI (kg/m²)	22.11 (20.75,24.22)	22.41 (21.03,22.41)	21.97 (20.42,24.01)	-1.394	0.163
Family history of diabetes,n (%)	10 (4.33)	6 (5.22)	4 (3.45)	0.114	0.736
Smoking exposure,n (%)	9 (3.90)	4 (3.48)	5 (4.31)	0.107	0.744
Alcohol consumption,n (%)	18 (7.80)	11 (9.57)	7 (6.03)	1.002	0.317
GDM	42 (18.18)	30 (26.09)	12 (10.34)	9.620	0.002
Male fetuses,n (%)	121 (52.38)	63 (54.80)	58 (50.00)	0.530	0.467
Mid-pregnancy					
FPG (mmol/L)	4.42 (4.18,4.68)	4.57 (4.32,4.82)	4.29 (4.08,4.51)	-5.098	<0.001
1h PG (mmol/L)	7.54 (6.68,8.65)	7.74 (7.00,9.00)	7.49 (6.33,8.35)	-2.630	0.009
2h PG (mmol/L)	6.75 (6.05,7.45)	6.86 (6.12,7.64)	6.63 (5.78,7.22)	-2.392	0.017
HOMA-β	191.53 (132.65,285.65)	175.27 (122.33,257.70)	207.16 (145.97,312.84)	-2.285	0.022
HOMA-IR	1.66 (1.29,2.25)	1.83 (1.31,2.61)	1.57 (1.22,2.02)	-2.828	0.005
TyG index	9.00 (8.80,9.25)	9.00 (8.82,9.29)	8.97 (8.74,9.23)	-1.671	0.095
TC (mmol/L)	5.63 (5.14,6.29)	5.55 (5.12,6.15)	5.72 (5.22,6.37)	-1.080	0.280
TG (mmol/L)	2.32 (1.87,2.90)	2.29 (1.88,2.90)	2.34 (1.84,2.89)	-0.160	0.873
HDL-C (mmol/L)	1.90 (1.72,2.13)	1.88 (1.71,2.13)	1.93 (1.74,2.14)	-0.529	0.597
LDL-C (mmol/L)	3.23 (2.87,3.61)	3.21 (2.83,3.50)	3.30 (3.00,3.66)	-2.049	0.040
SBP (mmHg)	113 (108,120)	113 (109,121)	112 (108,118)	-1.444	0.149
DBP (mmHg)	70 (65,76)	69 (62,76)	71 (66,76)	-1.985	0.047
Adverse pregnancy outcomes					
Pre-eclampsia,n (%)	8 (3.46)	6 (5.22)	2 (1.72)	1.192	0.275
Fetal growth restriction,n (%)	15 (6.49)	7 (6.09)	8 (6.90)	0.062	0.803
Preterm birth,n (%)	18 (7.80)	13 (11.30)	5 (4.31)	3.932	0.047
Macrosomia,n (%)	17 (7.36)	13 (11.30)	4 (3.45)	5.228	0.022
Neonatal respiratory distress syndrome,n (%)	7 (3.03)	4 (3.48)	3 (2.59)	0.156	0.693
Composite adverse pregnancy outcomes,n (%)	56 (24.24)	33 (28.70)	19 (16.38)	5.022	0.025

PDX1, pancreatic duodenal homeobox-1; GDM, gestational diabetes mellitus; BMI, body mass index; GDM, gestational diabetes mellitus; FPG, fasting plasma glucose; 1h PG, 1-hour postprandial blood glucose; 2h PG, 2-hour postprandial blood glucose; TyG index, triglyceride and glucose index; SBP, systolic blood pressure; DBP, diastolic blood pressure; TC, total cholesterol; TG, triglycerides; HDL-c, high-density lipoprotein cholesterol; LDL-c, low-density lipoprotein cholesterol.

### 3.3 Correlations of PDX1 in early pregnancy with glucose metabolic factors in mid-pregnancy

PDX1 in early pregnancy was negatively correlated with FPG (r=-0.320, P<0.001), 2h PG (r=--0.133, P=0.044), HOMA-IR (r=-0.179, P=0.007), and TyG index (r=-0.173, P=0.008) in mid-pregnancy, while positively correlated with HOMA-β (r=0.159, P=0.016). However, no correlation was found between PDX1 and 1h PG (**Table 3**). The scatter plot was further drawn in **Supplementary Figure 1**.

**Table 3:** Correlations of PDX1 in early pregnancy with glucose metabolic factors in mid-pregnancy.

Items	r	p
FPG	-0.320	<0.001
1hPG	-0.120	0.068
2hPG	-0.133	0.044
HOMA-IR	-0.179	0.007
HOMA-β	0.159	0.016
TyG	-0.173	0.008

FPG, fasting plasma glucose; TyG index, triglyceride and glucose index; 1hPG, 1-hour postprandial blood glucose; 2hPG, 2-hour postprandial blood glucose.

### 3.4 Association of PDX1 in early pregnancy and the risk of GDM

After adjusting for traditional risk factors (including age, preconception BMI, family history of diabetes, smoking exposure, and alcohol consumption), the logistic regression analysis revealed that PDX1 in early pregnancy was linked to a decreased risk of GDM (adjusted odds ratio [aOR] 0.287, 95%CI 0.130-0.636, P=0.002) (**Table 4**).

**Table 4:** Logistic regression analysis of PDX1 and GDM.

Items	β	SE	Wald χ2 value	OR	95% CI	P
Model 1						
Per 1 pg/mL	-0.021	0.009	4.954	0.979	0.092-0.998	0.026
PDX1<131pg/mL	Reference					
PDX1≥131pg/mL	-1.118	0.372	9.055	0.327	0.158-0.677	0.003
Model 2						
Per 1 pg/mL	-0.024	0.010	5.492	0.976	0.957-0.996	0.019
PDX1<131pg/mL	Reference					
PDX1≥131pg/mL	-1.248	0.406	9.466	0.287	0.130-0.636	0.002

Model 1: not adjusted; Model 2: adjusted for age, preconception BMI, family history of diabetes, smoking exposure, and alcohol consumption.

### 3.5 ROC curve analysis of diagnostic value of PDX1 and traditional factors in GDM

**Figure 2:**
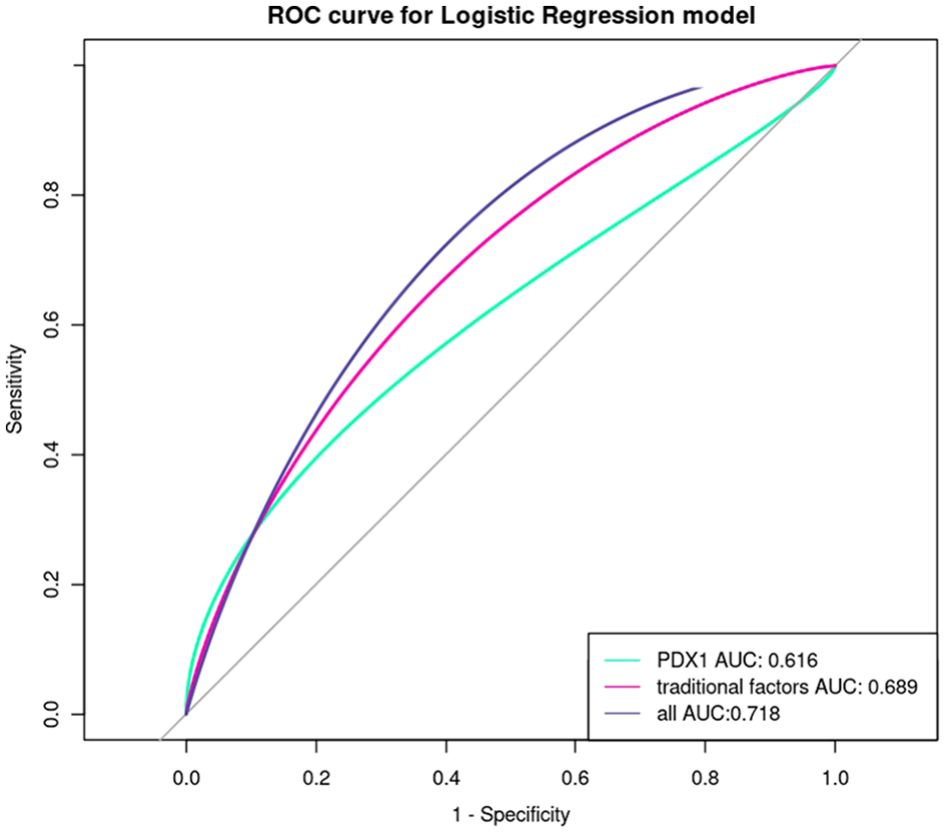
ROC curve analysis of the diagnostic value of PDX1 and traditional factors in GDM.

The area under the ROC curves (AUC) of PDX1 in early pregnancy for predicting the occurrence of GDM was 0.616 (P<0.05). The combination of PDX1 and traditional factors could improve the predictive value of GDM (AUC: 0.718, P<0.001, **Figure 2**, **Supplementary Table 3**).

### 3.6 Associations of PDX1 in early pregnancy with adverse pregnancy outcomes

After adjusting for traditional risk factors (age, preconception BMI, family history of diabetes, smoking exposure, alcohol consumption, and GDM), the logistic regression analysis showed that pregnant women with higher PDX1 levels in early pregnancy had a lower incidence of macrosomia (aOR 0.249, 95% CI 0.076-0.811, P=0.021) and composite adverse pregnancy outcomes (aOR 0.496, 95% CI 0.256-0.960, P=0.037, **Table 5**).

**Table 5:** Associations of PDX1 with adverse pregnancy outcomes.

Items	Model 1			Model 2		
	OR	95%CI	P	OR	95%CI	P
Pre-eclampsia						
Per 1 pg/mL	0.967	0.928-1.008	0.110	0.971	0.932-1.012	0.160
PDX1<131pg/mL	Reference			Reference		
PDX1≥131pg/mL	0.319	0.063-1.613	0.167	0.320	0.055-1.845	0.202
Fetal growth restriction						
Per 1 pg/mL	0.994	0.968-1.021	0.646	0.990	0.962-1.019	0.496
PDX1<131pg/mL	Reference			Reference		
PDX1≥131pg/mL	1.143	0.400-3.262	0.803	1.020	0.348-2.988	0.971
Preterm birth						
Per 1 pg/mL	0.974	0.948-1.001	0.059	0.971	0.942-1.000	0.049
PDX1<131pg/mL	Reference			Reference		
PDX1≥131pg/mL	0.353	0.122-1.026	0.056	0.326	0.104-1.021	0.054
Macrosomia						
Per 1 pg/mL	0.951	0.921-0.982	0.002	0.942	0.909-0.977	0.001
PDX1<131pg/mL	Reference			Reference		
PDX1≥131pg/mL	0.280	0.089-0.887	0.030	0.249	0.076-0.811	0.021
Neonatal respiratory distress syndrome						
Per 1 pg/mL	1.002	0.965-1.039	0.931	1.007	0.968-1.048	0.712
PDX1<131pg/mL	Reference			Reference		
PDX1≥131pg/mL	0.737	0.161-3.367	0.694	0.811	0.165-3.997	0.797
Composite adverse pregnancy outcomes						
Per 1 pg/mL	0.974	0.958-0.991	0.004	0.974	0.965-0.991	0.004
PDX1<131pg/mL	Reference			Reference		
PDX1≥131pg/mL	0.487	0.258-0.920	0.027	0.496	0.256-0.960	0.037

Model 1: not adjusted; Model 2: adjusted for age, preconception BMI, family history of diabetes, smoking exposure, alcohol consumption, and GDM.

## 4 Discussion

GDM has been shown to have serious negative impacts on the health of both mothers and infants. A recent study with 53,649 participants revealed that GDM is a significant predictor of adverse pregnancy outcomes, leading to various complications for both mothers and newborns (20). Early detection and treatment of GDM have been proven to be more effective and cost-efficient (21). Currently, GDM is diagnosed based on the results of OGTT conducted during the mid-pregnancy. Detecting and predicting GDM early, followed by prompt intervention, is crucial for reducing adverse pregnancy outcomes and improving the health of both mothers and infants. In this prospective study, we investigated the association of PDX1 in early pregnancy with GDM and adverse pregnancy outcomes. The results showed that elevated PDX1 levels in early pregnancy were associated with reduced risks of GDM (aOR 0.287, P=0.002) and composite adverse pregnancy outcomes (aOR 0.496, P=0.037). Besides, PDX1 in early pregnancy was negatively correlated with FPG, 2h PG, HOMA-IR, and TyG in mid-pregnancy, while positively correlated with HOMA-β (P<0.05).

GDM is a prevalent metabolic disorder initially diagnosed during pregnancy. This transient form of diabetes results from insulin resistance and pancreatic β-cell dysfunction. Normally, hormones produced by the placenta during pregnancy induce insulin resistance in the mother, ensuring adequate nutrient supply to the fetus. To maintain normal blood glucose levels, the maternal pancreatic β-cells must compensate by secreting more insulin (22). GDM develops when pancreatic β-cell function declines and cannot meet this increased demand. Late-stage pregnancy typically features maternal hyperinsulinemia and insulin resistance, which are especially pronounced in women with GDM (23). HOMA-IR and HOMA-β are considered to assess insulin resistance and pancreatic β-cell function with high accuracy (24). In this study, we also use HOMA-IR and HOMA-β to evaluate insulin resistance and pancreatic β-cell function of pregnant women, and found that patients with GDM had higher HOMA-IR in both early and mid-pregnancy, but had lower HOMA⁃β only in mid-pregnancy. The TyG index is also an indicator calculated using TG and FPG, that can be used to assess insulin resistance (2). Guo Y et al. found that the TyG index was proportional to the risk of GDM (aOR=2.10, P<0.001), and concluded that the TyG index in early pregnancy could predict GDM (25). Another study showed that except for TyG, high levels of FPG and TG in the first trimester were associated with an increased risk of GDM. In this study, patients with GDM had higher FPG, TG, and TyG index both in the first trimester and second trimester.

PDX1, also known as insulin promoter factor-1 (IPF1), somatostatin transcription factor-1 (STF1), or glucose-sensitive factor-1 (GSF1), is located on human chromosome 13q12.1 and consists of 6284 base pairs (26). PDX1 is a transcription factor primarily expressed in the pancreas, particularly in β-cells, where it plays a critical role in pancreatic development, β-cell differentiation, and the regulation of insulin gene expression. Before the maturation of the embryo, PDX1 is extensively expressed in both endocrine and exocrine cells. As the pancreas develops, PDX1 expression becomes predominantly localized to the β-cells of the mature pancreas (27). Therefore, it can be said that PDX1 is a symbol of β-cells identity. A study showed that in PDX1 knockout mice, their β-cells mature poorly after birth and the expression of several β-cells related genes was impaired (28). The protein encoded by PDX1 activates the transcription of several genes essential for regulating glucose metabolism, such as insulin, glucokinase, somatostatin, and pancreatic amylin (29). PDX1 also increases insulin secretion indirectly by activating the transcription and expression of glucokinase and glucose-transporter 2 (9). Several important nuclear proteins, including MafA, HMGA1, and NeuroD1, play pivotal roles in maintaining pancreatic β-cell function. Notably, PDX1 exhibits synergistic effects with both NeuroD1 and MafA in regulating insulin biosynthesis. A study has shown that coordinated expression of these three transcription factors significantly upregulates insulin gene expression, promotes insulin synthesis and secretion (30). Furthermore, HMGA1 can interact with PDX1 and MafA to enhance their transcriptional activation of the insulin gene promoter, thereby augmenting insulin production (31). Research reported that in adult pancreatic β-cells, short-term hyperglycemia enhanced the binding of PDX1 to the insulin gene, thereby increasing insulin mRNA levels. However, under the cytotoxic effects of prolonged hyperglycemia, both PDX1 and insulin levels decreased (32). In type 2 diabetes (T2DM), the expression levels of PDX1 are significantly compromised (33).

Considering the role of PDX1 in β-cell functionality, it is probable that PDX1 significantly contributes to the pathological process of GDM. Nasir I et al. discovered that prolactin could elevate the levels of PDX1 mRNA and protein in pancreatic islet cells of mice (34). A study showed that the high-fat diet during pregnancy in rats led to a significant reduction in PDX1 expression, damage to β-cells, and decreased insulin release (35). Furthermore, evidence from human studies further supports the close relationship between PDX1 and GDM. A study analyzing placental tissues from the fetal side demonstrated that the GDM group exhibited significantly reduced PDX1 mRNA expression levels compared to controls, and a negative correlation was observed between PDX1 mRNA levels and placental blood glucose levels (36). Additionally, another study investigated PDX1 mRNA expression in the peripheral blood of GDM patients and normal pregnant women. The results revealed that PDX1 mRNA expression was significantly lower in the GDM group (1.06 ± 0.18 vs. 1.35 ± 0.16, P < 0.05) and negatively correlated with neonatal blood glucose levels (r = −0.390, P = 0.013) (37). Our findings, which indicate lower serum PDX1 levels in GDM patients compared to those with normal glucose tolerance, are consistent with these studies. Although research on PDX1 expression in GDM remains limited, its role in other diseases has been explored. For instance, a study on pancreatic cancer utilized qRT-PCR to detect PDX1 transcripts in patient serum and reported significantly elevated PDX1 levels in pancreatic cancer patients compared to healthy controls (38). These findings suggest that PDX1 may serve as a potential biomarker across different pathological conditions, warranting further investigation into its role in GDM.

Under normal physiological conditions, PDX1 is a nuclear protein and is not secreted into the bloodstream (33). However, we found that PDX1 was present in serum in pregnant conditions. Firstly, hormones during pregnancy (such as prolactin) could elevate the levels of PDX1 mRNA and protein in pancreatic islet cells (34). Secondly, PDX1 might be secreted in extracellular vesicles (e.g., exosomes) under certain pathological stress or conditions (such as pregnancy). Thirdly, in cases of cellular stress, nuclear proteins like PDX1 could leak into the extracellular space and subsequently enter the bloodstream. Pregnancy is a special condition with stress and inflammation, during which PDX1 may present in serum. In this study, we found that the serum PDX1 levels in GDM patients were lower than those in women with normal glucose tolerance. This may be due to the damage to β cells caused by oxidative stress, inflammation, or autoimmune reactions, resulting in reduced release of PDX1. At the same time, there may be dysregulation of PDX1 gene expression, leading to decreased transcription or translation levels of PDX1, thereby reducing the release of PDX1 into the bloodstream. In addition, GDM patients may have impaired cellular secretion functions, resulting in decreased PDX1 secretion into the bloodstream via extracellular vesicles (such as exosomes). In this study, we also found that PDX1 levels were positively correlated with HOMA-β in pregnancy. HOMA-β serves as a crucial indicator for evaluating pancreatic β-cell function. Therefore, the levels of PDX1 might reflect the β-cell function in pregnancy.

This prospective cohort study first investigated the maternal serum PDX1 levels during pregnancy. The results showed that PDX1 in early pregnancy was negatively correlated with FPG, 2h PG, HOMA-IR, and TyG, while positively correlated with HOMA-β in mid-pregnancy. Moreover, the elevated PDX1 levels in early pregnancy were associated with reduced risks of GDM and adverse pregnancy outcomes. PDX1 had a modest predictive value for GDM. When PDX1 was incorporated into the predictive model for GDM, it slightly enhanced the predictive ability of traditional factors for GDM, but no significant statistical difference was observed (P > 0.05). Although the addition of PDX1 did not significantly augment the predictive value of conventional GDM risk factors, it offers a novel perspective for refining GDM prediction strategies.

Nevertheless, this study has several limitations. Firstly, the size of the sample was comparatively limited and exclusively drawn from an East Chinese population. We need a larger and more diverse sample to increase the persuasiveness of the findings. Secondly, we did not collect blood samples from pregnant women during childbirth, resulting in a lack of analysis of the complete trend of PDX1 throughout the pregnancy. Thirdly, we did not conduct follow-up monitoring after production, which prevented us from analyzing the long-term influence of PDX1 on the prognosis of GDM.

In summary, our results suggested that higher PDX1 levels in early pregnancy were associated with decreased risks of GDM and adverse pregnancy outcomes. It is suggested that PDX1 is significant for the early prediction of GDM and adverse pregnancy outcomes.

## Data Availability

The raw data supporting the conclusions of this article will be made available by the authors, without undue reservation.
