# Supplementary material for: PDX1 in early pregnancy is associated with decreased risks of gestational diabetes mellitus and adverse pregnancy outcomes
Source: Front Endocrinol (Lausanne). 2025 May 8;16:1486197. doi: 10.3389/fendo.2025.1486197 (PMC12094967; doi:10.3389/fendo.2025.1486197)
Supplement: Supplementary file 1 [file DataSheet1.docx]

| Supplementary Table 1. Comparison of metabolic factors between first trimester and second trimester in GDM and NGT groups | | | | | | | | | |
| --- | --- | --- | --- | --- | --- | --- | --- | --- | --- |
|  | GDM group | | | |  | non-GDM group | | | |
| Index | The first trimester | The second trimester | Z | P |  | The first trimester | The second trimester | Z | P |
| PDX1 | 123.21(108.38,139.78) | 81.65(71.34,92.75) | -5.545 | 0.000 |  | 132.15(119.35,143.10) | 96.77(77.43,111.56) | -10.703 | 0.000 |
| FPG | 4.75(4.47,5.23) | 5.12(4.66,5.41) | -1.616 | 0.106 |  | 4.63(4.42,4.88) | 4.36(4.15,4.59) | -7.234 | 0.000 |
| TG | 1.62(1.35,2.30) | 2.70(2.23,4.07) | -5.408 | 0.000 |  | 1.33(1.09,1.64) | 2.21(1.85,2.75) | -11.449 | 0.000 |
| TyG | 8.76(8.52,9.08) | 9.33(9.03,9.68) | -5.483 | 0.000 |  | 8.49(8.31,8.75) | 8.95(8.77,9.19) | -11.126 | 0.000 |
| HOMA⁃IR | 1.72(1.07,2.34) | 2.27(1.65,3.62) | -2.507 | 0.012 |  | 1.39(1.02,1.79) | 1.57(1.22,2.12) | -2.983 | 0.003 |
| HOMA⁃β | 110.26(83.52,188.32) | 148.97(112.21,209.16) | -1.557 | 0.120 |  | 114.42(83.82,156.86) | 198.59(140.82,297.90) | -8.336 | 0.000 |

| Supplementary Table 2. Comparison of metabolic indexes between male and female fetuses | | | | | | | |
| --- | --- | --- | --- | --- | --- | --- | --- |
| Index | All | Male group | Female group |  | Z/χ2 | *P* value |  |
|  | N=231 | N=121 | N=110 |  |  |  |  |
| **Early pregnancy** |  |  |  |  |  |  |  |
| FPG(mmol/L) | 4.65(4.42,4.92) | 4.63(4.42,4.91) | 4.65(4.44,4.94) |  | -0.463 | 0.643 |  |
| HOMA⁃β | 113.87(84.84,157.93) | 114.22(82.25,167.87) | 112.07(85.21,159.86) |  | -0.019 | 0.985 |  |
| HOMA⁃IR | 1.44(1.02,1.85) | 1.43(1.03,1.80) | 1.47(0.93,1.96) |  | -0.578 | 0.564 |  |
| TyG index | 8.52(8.36,8.79) | 8.51(8.33,8.74) | 8.57(8.37,8.86) |  | -1.284 | 0.199 |  |
| TC(mmol/L) | 4.38(4.03,4.94) | 4.38(4.04,4.89) | 4.35(4.01,4.95) |  | -0.298 | 0.766 |  |
| TG(mmol/L) | 1.36(1.14,1.74) | 1.36(1.14,1.64) | 1.39(1.17,1.89) |  | -1.375 | 0.169 |  |
| HDL-C(mmol/L) | 1.61(1.42,1.82) | 1.60(1.43,1.82) | 1.61(1.41,1.84) |  | -0.383 | 0.701 |  |
| LDL-C(mmol/L) | 2.45(2.18,2.85) | 2.44(2.21,2.75) | 2.46(2.15,2.92) |  | -0.529 | 0.597 |  |
| PDX1(pg/ml) | 131.15(118.29,142.96) | 125.18(113.77,144.48) | 131.90(115.26,149.83) |  | -1.211 | 0.226 |  |
| **Mid-pregnancy** |  |  |  |  |  |  |  |
| FPG(mmol/L) | 4.42(4.18,4.68) | 4.43(4.21,4.69) | 4.38(4.16,4.67) |  | -1.217 | 0.233 |  |
| 1h PG(mmol/L) | 7.54(6.68,8.65) | 7.48(6.60,8.54) | 7.70(6.47,8.85) |  | -1.411 | 0.158 |  |
| 2h PG(mmol/L) | 6.75(6.05,7.45) | 6.68(6.01,7.35) | 6.76(6.08,7.52) |  | -0.794 | 0.427 |  |
| HOMA⁃β | 191.53(132.65,285.65) | 191.53(127.07,284.23) | 190.76(134.85,290.36) |  | -0.565 | 0.572 |  |
| HOMA⁃IR | 1.66(1.29,2.25) | 1.71(1.31,2.23) | 1.63(1.23,2.31) |  | -0.179 | 0.858 |  |
| TyG index | 9.00(8.80,9.25) |  |  |  | -1.046 | 0.296 |  |
| TC(mmol/L) | 5.63(5.14,6.29) | 5.53(5.15,6.06) | 5.70(5.12,6.57) |  | -1.536 | 0.125 |  |
| TG(mmol/L) | 2.32(1.87,2.90) | 2.29(1.85,2.78) | 2.38(1.89,3.04) |  | -1.283 | 0.199 |  |
| HDL-C(mmol/L) | 1.90(1.72,2.13) | 1.89(1.72,2.14) | 1.92(1.73,2.13) |  | -0.435 | 0.664 |  |
| LDL-C(mmol/L) | 3.23(2.87,3.61) | 3.22(2.94,3.54) | 3.27(2.83,3.69) |  | -0.928 | 0.354 |  |
| PDX1(pg/ml) | 92.64(74.93,109.58) | 92.64(77.43,109.32) | 92.85(73.51,109.78) |  | -1.162 | 0.245 |  |
| **Adverse pregnancy outcomes** |  |  |  |  |  |  |  |
| Pre-eclampsia,n(%) | 8(3.46) | 3(2.48) | 5(4.55) |  | 0.247 | 0.619 |  |
| Fetal growth restriction,n(%) | 15(6.49) | 9(7.44) | 6(5.45) |  | 0.373 | 0.541 |  |
| Preterm birth,n(%) | 18(7.80) | 10(8.26) | 8(7.27) |  | 0.079 | 0.779 |  |
| Macrosomia,n(%) | 17(7.36) | 10(8.26) | 7(6.36) |  | 0.305 | 0.581 |  |
| Neonatal respiratory distress syndrome,n(%) | 7(3.03) | 3(2.48) | 4(3.64) |  | 0.016 | 0.898 |  |
| Composite adverse pregnancy outcomes,n(%) | 56(24.24) | 32(26.45) | 24(21.82) |  | 0.672 | 0.412 |  |

| Supplementary Table 3. ROC curve analysis of the diagnostic value of PDX1 and traditional factors in GDM | | | | | | | |
| --- | --- | --- | --- | --- | --- | --- | --- |
| Item | AUC | SE | 95%CI | P | Sensitivity | Specificity | Youden Index |
| PDX1 | 0.616 | 0.052 | 0.520-0.725 | 0.013 | 57.10% | 71.40% | 0.285 |
| traditional factors | 0.689 | 0.044 | 0.606-0.778 | 0.000 | 85.70% | 49.70% | 0.354 |
| all | 0.718 | 0.039 | 0.649-0.803 | 0.000 | 90.50% | 49.70% | 0.402 |


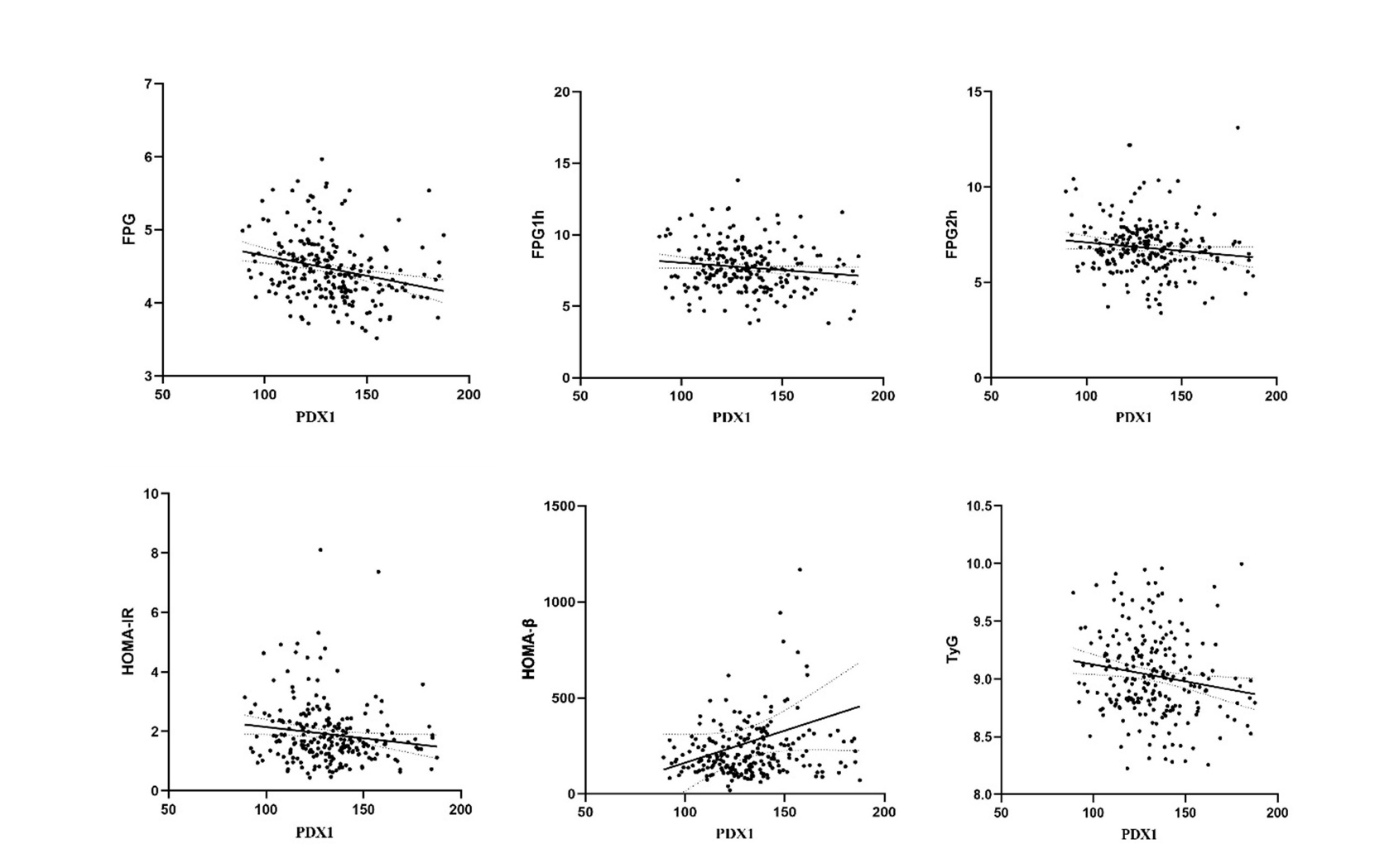


Supplementary Figure 1. The scatter plot of correlations between PDX1 in early pregnancy and glucose metabolic factors in mid-pregnancy
